# Chromosomal Sequence of Lactobacillus brevis Oregon-R-modENCODE Strain BDGP6, a Lactic Acid Bacterium Isolated from the Gut of Drosophila melanogaster

**DOI:** 10.1128/MRA.00931-20

**Published:** 2020-10-29

**Authors:** Kenneth H. Wan, Charles Yu, Soo Park, Ann S. Hammonds, Benjamin W. Booth, Susan E. Celniker

**Affiliations:** a Biological Systems and Engineering Division, Lawrence Berkeley National Laboratory, Berkeley, California, USA; Indiana University, Bloomington

## Abstract

Lactobacillus brevis Oregon-R-modENCODE strain BDGP6 was isolated from the gut of Drosophila melanogaster for functional host-microbial interaction studies. The bacterial chromosome is a single circular DNA molecule of 2,785,111 bp with a G+C content of 46%.

## ANNOUNCEMENT

The gut microbiome of *Drosophila* *melanogaster* is dominated by species from the genera *Lactobacillus* and *Acetobacter* (reviewed in references 1–3). *Lactobacillus* species produce vitamin B_2_, which is important for larval development (4, 5), increases amino acid metabolism, and promotes larval growth under conditions of nutrient scarcity (6). The first draft sequence of Lactobacillus brevis from *Drosophila* *melanogaster*, published in 2014, consisted of 117 contigs (3). We report here the complete bacterial chromosomal sequence, consisting of a single circular DNA molecule.

Lactobacillus brevis Oregon-R-modENCODE strain BDGP6 was isolated from the gut of Drosophila melanogaster as a single colony from lactobacillus MRS agar plates and cultured at 37°C for 16 to 18 h in MRS broth without shaking, and an aliquot was used for 16S V1 and V4 PCR (7) and sequence identification (reviewed in reference 8). DNA was isolated by cetyltrimethylammonium bromide (CTAB)/NaCl and phenol-chloroform extractions, followed by cesium chloride banding and isopropanol precipitation (9); then, it was sent to the National Center for Genome Resources (NCGR; Santa Fe, NM) for whole-genome sequencing using Pacific Biosciences (Menlo Park, CA) long-read sequencing on the RS II instrument (10). A single-molecule real-time (SMRT) cell library was constructed with 5 to 10 μg of unsheared DNA, size selected to >7 kb (BluePippin, Sage Science) using the PacBio 20-kbp protocol, and sequenced on one SMRT cell using P6 polymerase and C4 chemistry with 6-h movie times. Quality control filtering was performed via the PacBio SMRT Analysis Portal with smrtanalysis_2.3.0.140936.p5.167094 software. Default parameters were used for all software tools, except when otherwise noted.

Sequencing yielded 22,365 reads totaling 319,828,877 bp (chromosomal coverage, >70-fold), with a filtered mean read length of 14,300 bp and an *N*_50_ value of 20,694 bp. A *de novo* assembly was constructed using the HGAP2 protocol from SMRT Analysis v2.0 (11, 12). The final contig was manually trimmed and reviewed to produce a single circular chromosome. The sequence quality was assessed using minimap2 (13). The percentage of reads mapping to the assembly is 82.84%, and the estimated coverage is 72.58×. The remaining reads map to likely plasmids not characterized here. Annotations were predicted using the RAST v2.0 (annotation scheme, ClassicRAST) tool (14) and the NCBI Prokaryotic Genome Annotation Pipeline (PGAP) v4.2 (15).

The PGAP annotation predicted 2,676 protein-coding genes, 60 pseudogenes, 5 rRNA operons, and 71 tRNAs, 70 with canonical anticodon triplets and 1 undetermined (tRNA-OTHER). Of the 2,676 protein-coding genes, 332 are contained within candidate prophages. Like other L. brevis strains, BDGP6 contains integrated likely prophages. There are nine copies, namely, five complete, ranging in size from 38,511 to 49,964 bp, and four partial copies, ranging in size from 4,871 bp to 13,432 bp. Together, the prophage regions constitute 8.6% of the genome sequence. At the nucleotide level, they have little sequence similarity except for two copies that share 92% similarity (BLASTN v2.0MP-WashU) (16) for about 10 kb. Two cornerstone proteins highly conserved in prophages, the large terminase subunit and the portal protein (reviewed in reference 17), are encoded by four complete prophage copies. Based on its growth in MRS broth supplemented with antibiotics, strain BDGP6 is resistant to tetracycline (20 μg/ml), spectinomycin (50 μg/ml), kanamycin (50 μg/ml), gentamicin (20 μg/ml), and chloramphenicol (0.5 μg/ml), likely aided by 39 genes encoding ribosomal protection, efflux transporter, and antibiotic-modifying proteins. KEGG Automatic Annotation Server (18) pathway analysis showed that the genome encodes 56 enzymes involved with B vitamin metabolism and biosynthesis. In addition to vitamin B_2_ (riboflavin), L. brevis BDGP6 encodes a vitamin B_5_ (pantothenate) transporter, a cystine transporter (cystine is converted to two cysteines under high pH), and the biosynthetic pathway (five genes encoding the enzymes pantothenate kinase [CoaA], coenzyme A [CoA] biosynthesis bifunctional protein [CoaBC], phosphopantetheine adenylyltransferase [coaD], and dephospho-CoA kinase [coaE]) to convert these two molecules into CoA, an essential component of fatty acid synthesis and energy production.

### Data availability.

The chromosome sequence of Lactobacillus brevis Oregon-R-modENCODE strain BDGP6 is available under the GenBank accession number CP024635. The sequencing reads are available under the SRA accession number SRR12450050.
